# Heredity and the Race

**Published:** 1895-12-14

**Authors:** 


### Heredity and the Race.

Even the least scientific among us is willing to admit the influence of heredity. However much people may gird against predestination, and however strongly they may maintain that each individual is free to shape his movements and to decide his course in every circumstance in life, yet it is not easy to gainsay the old scriptural dogma that no man by thought can add a cubit to his stature. Whence, then, came his stature? Was it an accident, or was it a freak? Or was it not rather from his parentage? That it was the latter we all admit. However much we may differ as to the motive of man's actions, no one doubts about the origin of his bodily peculiarities. In height, in colour, in general build he follows his family, while even his peculiarities are traceable to one or other of his forbears. As regards all these things we have no doubt, and when we go into the stock yard or the kennel, or when we discuss the matter with the horse breeder, the poultry fancier, or the cattle raiser, we find universal and unanimous testimony that no animal is responsible for his own structure, but that in form, in endurance, in temper, and in longevity he is the product and representation of those which have gone before.

These things are granted. But how they happen is another matter. It certainly is not true that all offspring are like their parents, and even when the general likeness may be striking, the points of difference may be even more striking still; and thus, in studying the causes of that general family resemblance which tells us at once the breed of sheep or the strain of poultry, and makes it clear that our friend is a chip of the old block, we have also to inquire how it is that the shepherd knows the different sheep which form his flock—how we tell one brother from another. The study of variation is then quite as important as that of heredity. Putting the matter crudely, we have to decide whether the variation is the result of varying surroundings during development, or of varying degrees of the different parental tendencies implanted in the ovum from the beginning. In other words, are variations due to influences affecting growth, or to inherent tendencies derived from ancestors, and existing in different ova in varying degree? Interlocked with this is another question—are the tendencies and peculiarities which the parent may have developed during his life transmitted to his offspring, or are the tendencies which are transmitted only the sum of those which are derived from parental ancestors on both sides? No one need pretend that this is a trivial question. If the ovum can be influenced for good or ill by its surroundings, and if every impress made upon it or upon the growing child or the developing youth is to have effect not only upon this generation but on its descendants, then is our responsibility great indeed. Those who permit women with child to work in factories, who allow infants to be reared amid unhealthy surroundings, who suffer them to be brought up on improper food, who send imperfectly developed youths to work at injurious trades and to become "set" in abnormal shapes and postures, are not merely injuring a certain number of suffering individuals, but are impressing an ineradicable stamp of degeneration upon the future of the race. On the other hand, if all these terrors are unreal, if what is transmitted is what is inherited and not what is acquired, our course is clear, and is, moreover, pleasant. We have but to marry healthy wives of good feature, and good family, and our duty to posterity is done.

The biologist sees two possible ways of looking at the great mystery of the progression of life upon the globe. That from one generation to another life is transmitted by means of what one may call germinal cells, cells which have the power of producing again a more or less similar being, is clear enough. But while some would say that these cells are but representative of the sum total of the bodily powers of the individual who produces them, that in fact the germ is the product of the man, others would maintain that the germ in its development surrounds itself with the great body of the man in order that it, the germinal matter, may be nourished and well fed; and that so far from the germ being representative of the man with all his acquired vices, the man is the mere supporter of the germ, a mere food supplying mechanism, and that this germinal matter remains endowed with all its powers for good or ill irrespective of what happens to the animal in whose body it is borne for that particular generation.

If this latter view is to be accepted, it is no doubt a serious blow to those who think that by their own good deeds they can become regenerators of the race. For they can do nothing of the kind. The most virtuous living may result in a puny infant, while the wildest rake may be the father of an Apollo. Galton says, "The more bountifully a parent is gifted by Nature, the more rare will be his good fortune if he begets a son as richly endowed as himself, and still more so if he has a son who is endowed more largely."

On the other hand, it must be remembered that the tendency to revert to the average of many ancestors may well relieve the anxiety of those who fear that their children will inherit their own weaknesses and vices. It is the average of the ancestors which is of chief importance. Very tall people, very witty people, people of extraordinary mental capacity —nay, even very beautiful people—are often sports, oddities, and abnormal products of very common stocks, and the offspring of such people tend to revert to the average of their ancestors; while others not quite so tall, so witty, or even so beautiful, may, if they are but average representatives of their families, tend to breed true and produce their like.

To the anxious reformer, who hopes by his tiny efforts to make some impress on the world's history, Weismann's theory of heredity is a knockdown blow; but to the humble-minded man, who sees the world as it is, with all its sordid want and wickedness, it is full of hope, for it shows how the human race may, without serious degeneration, live through its time of trial, even through many ages of degradation, and yet may ultimately revive.

